# Exploring shape diversity and sexual dimorphism in two populations of *Nigma conducens* through geometric morphometrics

**DOI:** 10.1186/s40850-025-00224-4

**Published:** 2025-02-08

**Authors:** Safa M. El-masry, Tarek G. Ismail, Asmaa N. Mustafa

**Affiliations:** Group of Invertebrates Ecology - Department of Zoology, Faculty of Science, Sohag University, Sohag, 82524 Egypt

**Keywords:** Spiders, Geomorphics morphometrics, Shape variation, Sexual dimorphism, Allometry

## Abstract

**Background:**

Spiders are highly adaptable hunters found in nearly all terrestrial ecosystems and play an important role in biological control by preying on pest insects. Spiders’ body size and shape are vital for their survival, particularly in prey capture, and these morphological features are often utilized in cladistic analyses. This study employed geometric morphometrics to investigate prosoma shape and size variations between two populations of *Nigma conducens* spiders and between sexes within each population. Principal Component Analysis (PCA) explored shape variation, while Canonical Variate Analysis (CVA) compared shape differences between populations and sexes. Multivariate regression analysis was used to check for allometry.

**Results:**

MANOVA results revealed significant shape variations in spider prosoma between the two populations and between sexes, though the degree of these differences was small. The considerable overlap in individual shapes between populations may indicate a response to microhabitat similarity. Additionally, sexual dimorphism was observed in the prosoma shape of *N. conducens*, likely due to sexual selection or adaptive divergence related to different microhabitats. Size differences between sexes were insignificant in either population, particularly in the first, suggesting that prosoma size does not contribute to reproductive success. Moreover, the non-allometric relationship indicated that shape variations between the populations were independent of size.

**Conclusion:**

Overall, these findings highlight the complexity of morphological adaptations in *N. conducens* in response to ecological pressures and sexual selection.

**Supplementary Information:**

The online version contains supplementary material available at 10.1186/s40850-025-00224-4.

## Background

The homogeneity of the environment, where an animal lives, creates little selective pressures that may lead to less pronounced phenotypic differences among individuals. Conversely, some environments may exert strong selective pressures on certain physical traits, leading to variations in these traits between individuals of the same species [1, 2]. These variations in morphology depend on the phenotypic response to selective environmental pressures, allowing for better environmental adaptations [3, 4]. This is known as phenotypic plasticity, and it will enable individuals to adjust their phenotype in response to changes in their environment.

Several studies have indicated a relationship between individuals’ phenotypes and where they choose to live [5, 6]. Individuals of populations while dispersed tend to prefer similar habitats, enabling them to speed their adaptation to their surroundings [7]. The decision on where to live can be influenced by genetics, shaping an individual’s habitat choice. This process, known as matching habitat choice,’ involves individuals selecting environments that align best with their traits [6, 8].

In this regard, morphometrics is a valuable method for analyzing variations in body shape and sexual dimorphism [9, 10]. The analysis of morphometric data allows for identifying discrete patterns within continuous data [11–13]. Additionally, the landmark technique, one of the geometric morphometric methods (GMM), stands among the suite of analytical tools successfully applied to the analysis of general shape and sexual shape dimorphism [11, 14–19].

Also, allometry, the study of size-related shape variations, is an important factor in morphological variability among individuals or sexes [3, 20]. Allometry falls into three categories: ontogenetic allometry, which relates to changes in shape associated with size during development; static allometry, which examines the covariation between size and shape at a specific developmental stage within a population; and evolutionary allometry, which investigates the covariation between size and shape across populations [3, 20]. Allometric analysis combines the geometry of the data, mathematical deformations, and biological interpretations of shape variation [21]. Additionally, allometry analysis plays a key role in accounting for a large portion of morphological variation [22].

Spiders are incredibly adaptable predators that can be found in almost every land-based ecosystem, except for Antarctica. Because of this, they have been extensively researched in various fields of study, such as ecology and animal behavior. Morphometrics has been broadly applied to the Araneae, focusing on the genitalia, carapace and legs of different spiders to identify species or genera [13, 23–28]. Also, GMM methods were applied to spiders for identification purposes using ocular patterns and female genitalia [28–30], for sexual dimorphism using chelicera, forelegs and palp [26, 31]. In addition, shape-allometry variations and the effect of insecticides on shape variation were investigated [32].

Among all terrestrial groups, spiders stand out as having a noticeable prevalence of smaller males. Typically, in most spider species, females tend to be significantly larger in size compared to males which may reach > 12 times of males [33]. The members of the family Dictynidae constitute a widespread small to medium-sized, cribellate spiders which make irregular webs. *Nigma*, a genus of the family Dictynidae, is mostly plant dwellers and is found on the foliage of trees [34]. The genus *Nigma* contains 14 + species across various regions including North America and Northern Africa. *Nigma conducens* (O. Pickard-Cambridge, 1876) are small-sized spiders up to 5 mm in length and were collected from Egypt [35, 36].

There is little work on *Nigma conducens*, however, the published work focuses on its taxonomy, occurrence, distribution, and relationship with vegetation as its microhabitat [37, 38]. The size and shape of individuals within the species show a remarkable degree of consistency, with both males and females displaying nearly similar morphological traits. This characteristic makes *Nigma conducens* an excellent model organism for investigating the effects of microhabitat homogeneity/heterogeneity on morphological variation. Additionally, it offers a unique opportunity to study the role of sexual dimorphism in shaping size and shape differences within the species. Given its small size, distinct morphological traits, and ecological relevance, *Nigma conducens* hold significant potential for advancing our understanding of arachnids’ ecological and evolutionary processes. Therefore, the current study aims to (i) Examine and describe morphological changes in prosoma shape in two populations of *Nigma conducens* as a response to microhabitat characteristics to gain insights into the phenotypic plasticity or adaptation and evolution of spiders to their environment, (ii) Investigate how intraspecific allometry influences shape variations observed within the species, and (iii) Examine sexual dimorphism based on the variations in size and shape of the prosoma to characterize them as an indicator of secondary sexual differentiation. Also, with the presence of the River Nile as a barrier, the distribution of this species offers a significant opportunity to examine the impact of geographic barriers.

## Materials and methods

### Sites of collection and sampling

**Fig. 1:**
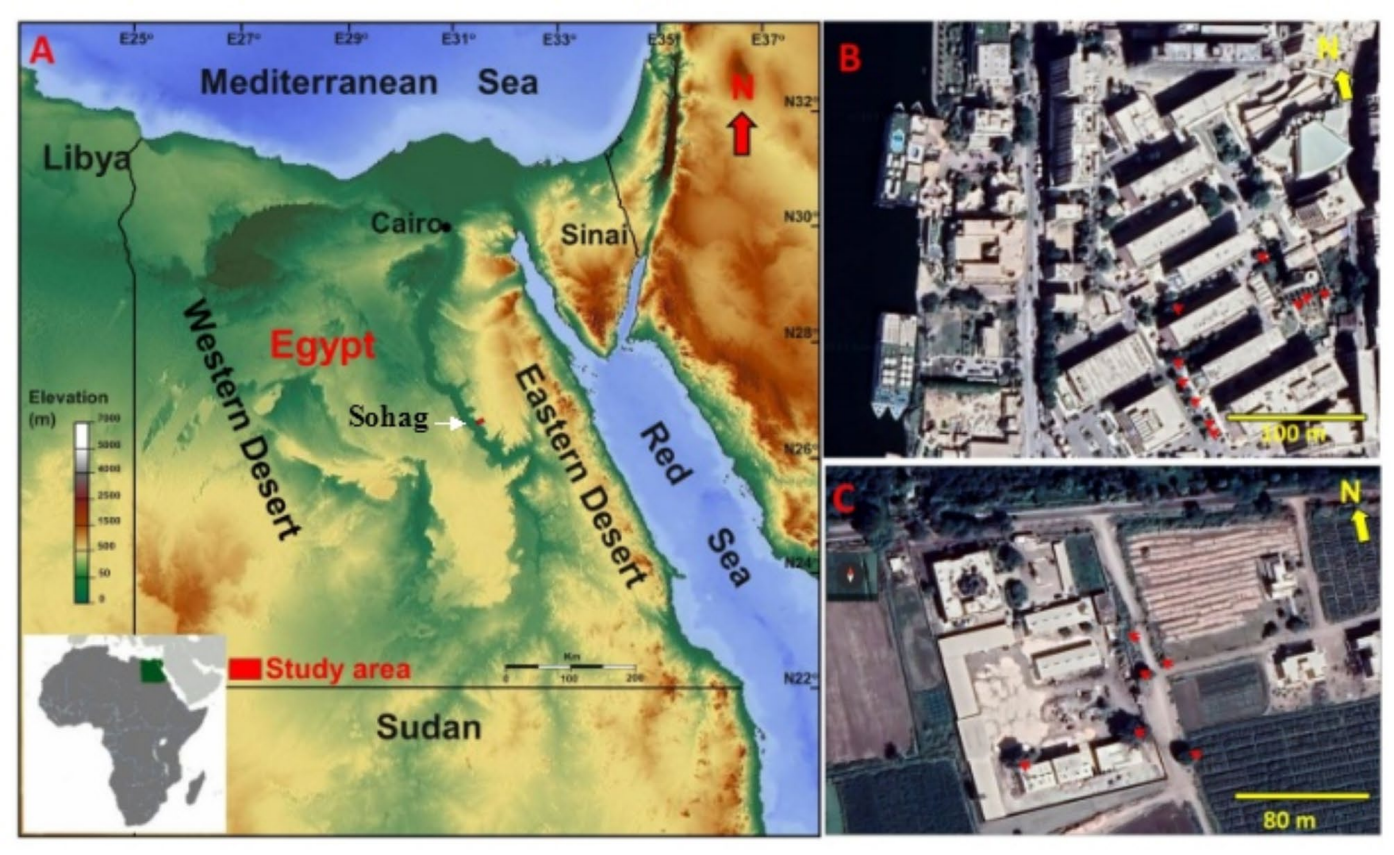
Map of Egypt showing the location of Sohag Governorate and sites of collection. To the upper right a Google earth map showing the first site, campus of Sohag University. While at the lower right a Google earth map showing the second site, Agriculture Research Center at Jazirat Shandweel. Red arrows refer to the trees, where the specimens were collected

Sohag is one of the Upper Egypt Governorate that locates between 26° 54’ 15” N, 31° 24’ 15” E, and 26° 11’ 55” N, 32° 04’ 22” E (Fig. 1A). In this study two sites, located 13.4 km apart, were selected to represent urban and agricultural settings. The first site, the campus of Sohag University, is situated on the eastern bank of the Nile River and lies within an urban area. Its coordinates range from 26°33’48.4"N to 26°33’58.4"N latitude and 31°42’25"E to 31°42’32.9"E longitude. The second site, Jazirat Shandweel Agriculture Research Centre (JSARC), is located north of Sohag city on the western bank of the Nile River and represents an agricultural setting. Its coordinates range from 26°37’41.65"N to 26°38’7.59"N latitude and 31°38’47.44"E to 31°39’37.95"E (Fig. 1B, C). The Sohag University campus is exclusively populated with ornamental trees, while JSARC contains ornamental trees surrounding orchard trees. For comparative purposes, only ornamental trees were selected from both sites.

Within each site, a specific tree habitat, *Ficus nitida* at the first site and *Dalbergia sissoo* at the second site were chosen to collect the investigated spider species. The two tree types constitute more than 90% of the ornamental trees in both sites. In addition to the geographical distance between the two sites, the River Nile separates them as a natural barrier.

Spider samples were obtained from two tree types by gathering the spider-hosting leaves (microhabitats) and keeping them in plastic containers. In the laboratory, the leaves were further inspected under a stereomicroscope and spiders were extracted from their webs by hand or with a fine needle. The spiders were then classified by sex, with females identified by their genital plate, while males were identified by their pedipalps. One leaf can hold one to a few individuals, however, females and males are never present on the same leaf.

The collected spider species are identified using the following keys [39–41]. A total of 129 undamaged individuals were selected for the analysis. Of these, 70 were females (28 from *F. nitida* trees and 42 from *D. sissoo* trees), and 59 were males (27 from *F. nitida* trees and 32 from *D. sissoo* trees).

### Microhabitat features

**Fig. 2:**
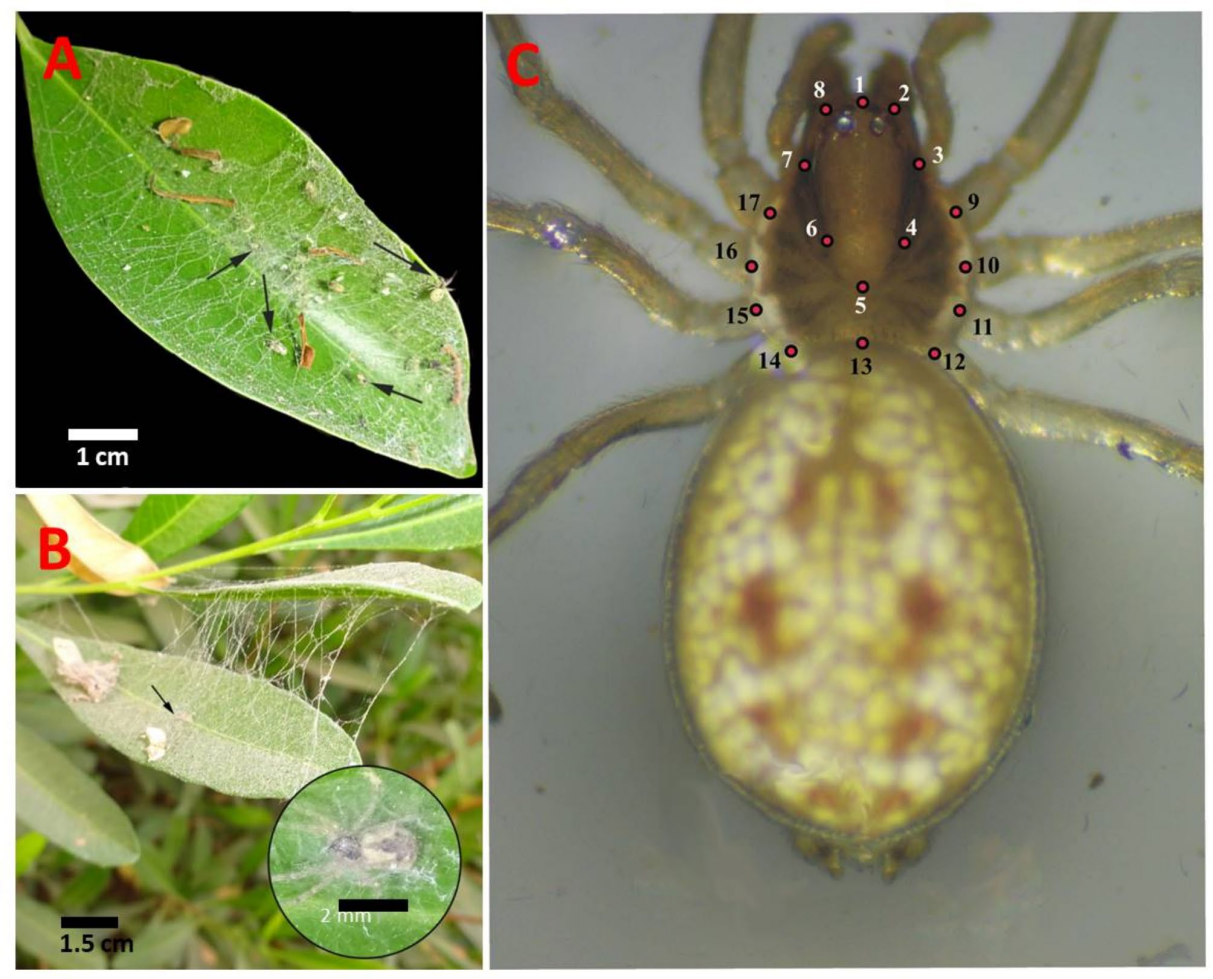
**A** and **B**, showing specimens of *Nigma conducens* spider in their natural habitat resting in their webs. **C**, showing the location of 17 morphological landmarks on the prosoma, which is used in the geometric morphometric analysis

The spider *Nigma conducens* is typically found on the upper surfaces of *Dalbergia sissoo* and *Ficus nitida* leaves. In these microhabitats, it constructs its distinctive, irregular, and finely meshed webs (Fig. 2A, B).

Both tree species are prevalent in Egypt, capable of reaching heights of up to 25 m, and have broad canopies with dense foliage. *Dalbergia sisso* is valued for its fragrant, decorative wood and ornamental appeal. It enhances landscapes while serving ecological (esteemed for its high-quality wood), aesthetic roles, and traditional medicinal uses. *Ficus nitida*, also, is a popular ornamental tree known for its attractive foliage and versatility in landscaping. It enhances the aesthetic appeal of various environments. *Dalbergia sissoo* and *Ficus nitida* are very similar to each other, however, they show few variations regarding their leaves (Table S1, Fig. S1).

### Geomorphic morphometric analysis

#### Data acquisition

Collecting data involved capturing images of the dorsal view of the prosoma using a digital camera (Axiocam ERc 5s) mounted to a binocular zoom stereo-microscope (Zeiss, Stemi 350). The two parts of the spider’s prosoma were analyzed using seventeen anatomical landmarks, identified with the tpsDig 2.22 software [42]. Landmarks 1–8 correspond to the pars cephalica, while landmarks 9–17 represent the pars thoracica (Fig. 2C). The abdomen was not considered in this study due to its softness and varying size, where its size and shape are function of foraging success and reproductive state in females and used up reserves in males [43].

Specimens were organized into two datasets to cover the objectives of the present investigation and labeled as “microhabitat” and “sex”. Each specimen was photographed twice, and each photo was digitized twice on four different sessions by the same author, therefore, image and digitizing errors were assessed.

### Size analysis

The spider prosoma length and width (in mm) were recorded for studied specimens of two populations. Also, the centroid size (CS) was used as a proxy to measure the overall size of the spiders. This measurement is commonly used in geometric morphometrics to assess an object’s size [12]. The centroid size is determined by taking the square root of the total of the squared distances between each landmark and the body centroid. In this study, CS was log-transformed for linear comparisons [44]. One-way ANOVA was used to analyze the size variations of spiders in different microhabitats and between sexes.

### Shape analysis

Landmarks from all specimens in each dataset were analyzed using a generalized Procrustes analysis (GPA). GPA aligns all configurations to eliminate variations (non-shape effects) of orientation, scale, and position, allowing for calculating the Procrustes average shape. This was followed by checking outliers’ mistakes in the landmarking and correcting them. Once the non-shape variations were removed, only geometric information related to shape remained, referred to as shape variables (or shape effects).

Principal Components Analysis (PCA), Discriminant Function Analysis (DFA), and Canonical Variate Analysis (CVA) were conducted in the present work. PCA is exploratory because it provides insights into the shape similarities or differences. PCA was performed on the variance-covariance matrix of the two datasets to reduce data and generate new shape variables (PC scores) that facilitated the exploration of the relative relationships between individual shapes.

CVA analysis (using Procrustes aligned shape data) is performed to maximize the separation between spiders in different microhabitats and between sexes relative to shape variation within groups. On the other hand, DFA analysis is applied to assign spider individuals to their correct population or sex based on shape features. The accuracy of classifying microhabitats and sexes was demonstrated through DFA by utilizing Mahalanobis distances in conjunction with a permutation test that included 10,000 randomizations. The classification results were validated through the Jackknife technique to test the effectiveness of categorizing the specimens into their respective groups. A multivariate analysis of variance (MANOVA) was conducted on PC scores of each dataset to determine whether there were differences in spider prosoma shapes across different microhabitats, and between sexes. PC scores accounting for more than 90% of the total variance were used as dependent variables, while microhabitats and sexes served as independent variables.

### Allometric trajectories

To examine how differences in size affect variations in shape, multivariate regression utilizing the combined variance within groups was used to study shape allometry [45]. Thus, multivariate regression analyses were conducted on shape (using Procrustes coordinates as the dependent variables) and size (using log centroid size as the independent variable) [46]. The independence between shape and size was analyzed through a permutation test with 10,000 runs [46, 47]. Then, a multivariate analysis of covariance (MANCOVA) was used to compare allometric trends across different datasets (microhabitat and sex) utilizing the TPSRegI 1.45 software [42]. The slopes of allometric trends were compared using the test for common slopes which assessed the significance of the interactions between “microhabitat X size” and “sex X size [48]. The significance of this interaction implies that allometric trends between the two datasets are not aligned, showing different directions. In the case of insignificant interaction, a second multivariate analysis was conducted by excluding the interaction (i.e., controlling the effect of allometry). The significance of the variables (microhabitat or sex) means that microhabitat and/or sex have parallel allometric trajectories (with identical slopes) and share a common allometric trajectory which may explain size-related shape differences [48].

Sexual dimorphism was illustrated through the differences in the average shape of the prosoma between females and males. The statistical analyses were conducted using the PAST (V. 3.26) software [49] and the SPSS software [50]. Additional geometric morphometric analyses, including GPA, outlier detection, Procrustes ANOVA, PCA, DFA, and multivariate regression analysis, were carried out using the MorphoJ integrated package (V. 1.07a) [46]. Corel Draw software was utilized to redraw figures for improved visualization.

## Results

### Measurement errors

The Procrustes ANOVA analysis shows negligible digitizing errors for shape variations compared to individual variations in the two populations of spider *Nigma conducens*. This was confirmed by Pillai’s trace analysis (Table S2).

### Size variations

**Fig. 3:**
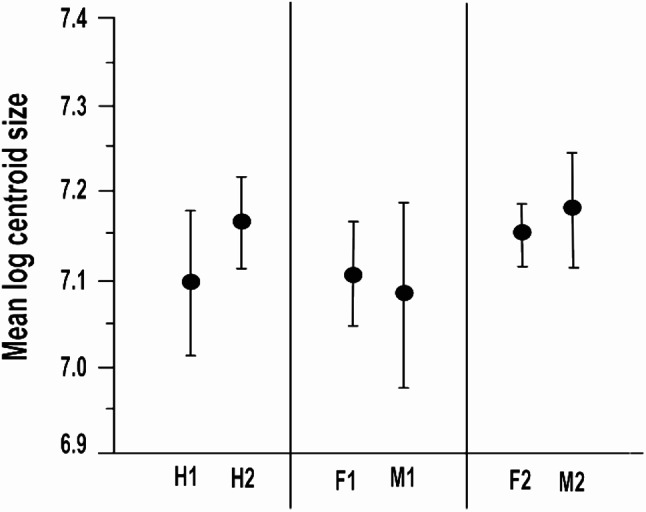
Comparison of size variation between two populations and between the two sexes within each population. Size is expressed as the mean ± SD of log centroid size. H1 = first population, H2 = second population, F and M refer to females and males, respectively

Traditional measurements showed that, at the first site, spider prosoma length ranged from 1.21 to 1.85 mm, with an average of 1.50 ± 0.11 mm. The prosoma width varied from 1.07 to 1.49 mm, with an average of 1.29 ± 0.09 mm. Comparatively, in the second site, prosoma length ranged from 1.38 to 1.82 mm (average 1.62 ± 0.1 mm), while width ranged from 1.17 to 1.54 mm (average 1.37 ± 0.07 mm). ANOVA analysis showed that the spiders of the second population had somewhat larger prosoma than those of the first one (length: F = 13.01, *P* < 0.05; width: F = 32.15, *P* < 0.001). Additionally, centroid size (measured as log CS) shows that the mean prosoma size of the second population of *Nigma conducens* spiders tends to be larger than that of the first population (F = 30.64, *P* < 0.001) (Fig. 3).

Regarding the size difference between sexes in each microhabitat, traditional measurements showed that males were slightly longer than females in the second microhabitat (F = 4.3, *P* = 0.04), but both sexes have equal widths (F = 0.20, *P* > 0.1). In comparison, the sexes of the first microhabitat were nearly the same length and width (length: F = 2.1, *P* = 0.14; width: F = 0.23, *P* = 0.63). Geometrically, there was no significant difference in size between sexes within each population, particularly in the first one indicating no size sexual dimorphism (H1: F = 1.57, *P* = 0.23; H2: F = 3.38, *P* = 0.07).

### Shape variations

**Fig. 4:**
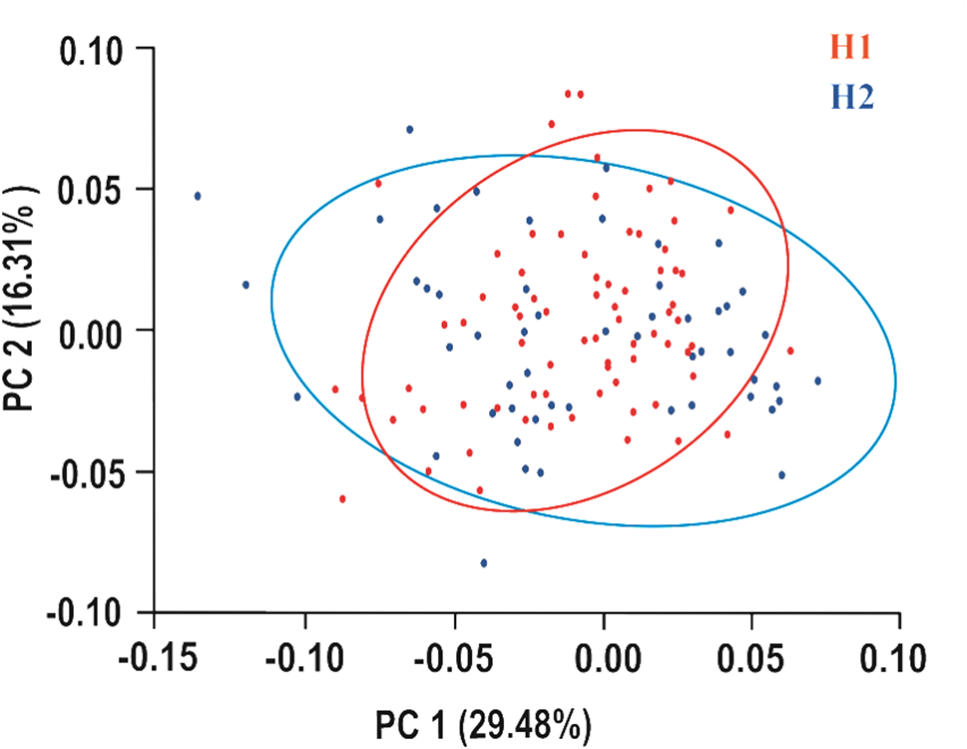
PCA analysis of shape variations between two populations. The scatter plot of principal component (PC) scores of the prosoma shape of the spider *Nigma conducens* reveals the overlap in the distribution of individual shapes in morphospace. H1 = first population, H2 = second population

**Fig. 5:**
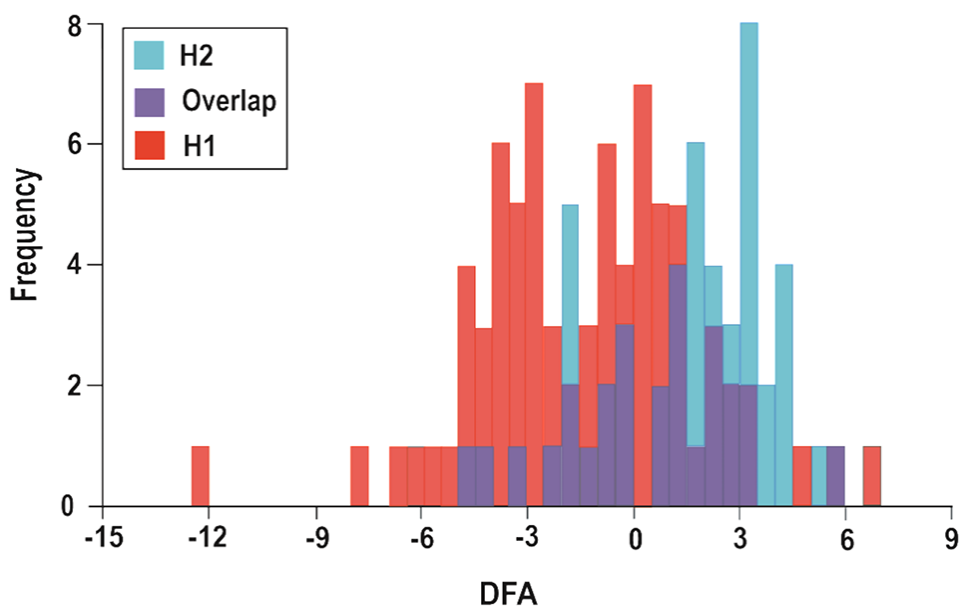
Discriminant function analysis (DFA) showing how habitat characteristics affect the shape of individuals from two populations. The frequency histogram reflects the overlap in shape between the two populations. H1 = first population, H2 = second population

The MANOVA analysis showed significant variations in the shape of the prosoma between the two spider populations (Wilks λ = 0.5, F _(30,96)_ = 3.26, *P* = 0.000). PCA illustrates overall variation in the distribution of the two populations within the space defined by the first two principal components and highlights their overlaps (Fig. 4). The analysis revealed that the first 13 axes account for 90% of the total shape variability between two populations. PC1 (29.48%) and PC2 (16.31%) accounted for more than 45% of total variance. Discrimination functional analysis (DFA) highlighted some shape variations as indicated by Hotelling’s *T*^*2*^ test (F = 127.07, *P* < 0.001). However, both PCA and DFA show an overlap between the two populations (Mahalanobis distance = 2.01; Procrustes distance = 0.017, *P* = 0.128 after permutation tests) (Figs. 4 and 5). DFA correctly classified 83.9% of individuals to their population, whereas correct classification was dropped to 68.8% after cross-validation analysis (Table 1).

**Table 1:** Overall classification of the two populations of *Nigma conducens* spider according to cross-validation analysis

Predicted Group Membership			
Sites	H1	H2	Total
H1	15(27.3%)	40(72.7%)	55(100%)
H2	49(66.2%)	25(33.8%)	74(100%)

H1 and H2 referred to the two populations

83.9% of original individual cases were correctly classified

68.8% of cross-validated grouped cases were correctly classified

**Fig. 6:**
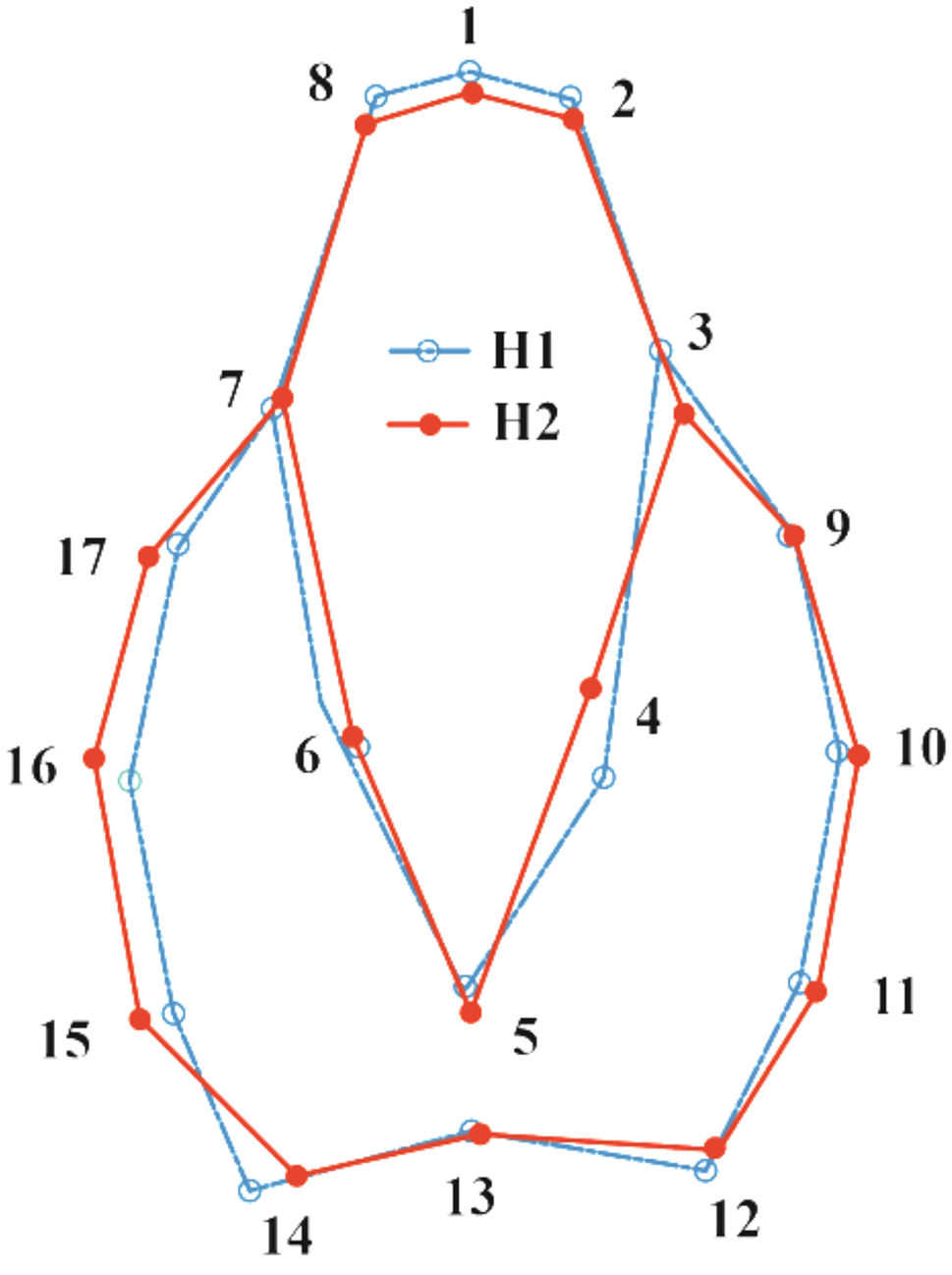
Wireframe drawings representing the deformation of prosoma shape based on DFA between the two populations of the spider *Nigma conducens*. H1 = first population, H2 = second population. (5X magnification to show group differences)

The shape variations reflected a slight protruding of the cephalic region and narrowing of the lateral sides of prosoma in the individuals of the first population. On the other hand, the individuals of the second population showed marked expansion of the prosoma lateral sides and a slight reduction of the cephalic region (Fig. 6).

### Effect of size on shape for two populations (allometry)

The multivariate regression analysis revealed a non-effect of size on shape among the populations (Wilks’ λ = 0.492; F = 1.10 *P* = 0.37). Size explains only 1.2% of the variation in prosoma shape between the two populations.

### Shape sexual dimorphism

**Fig. 7:**
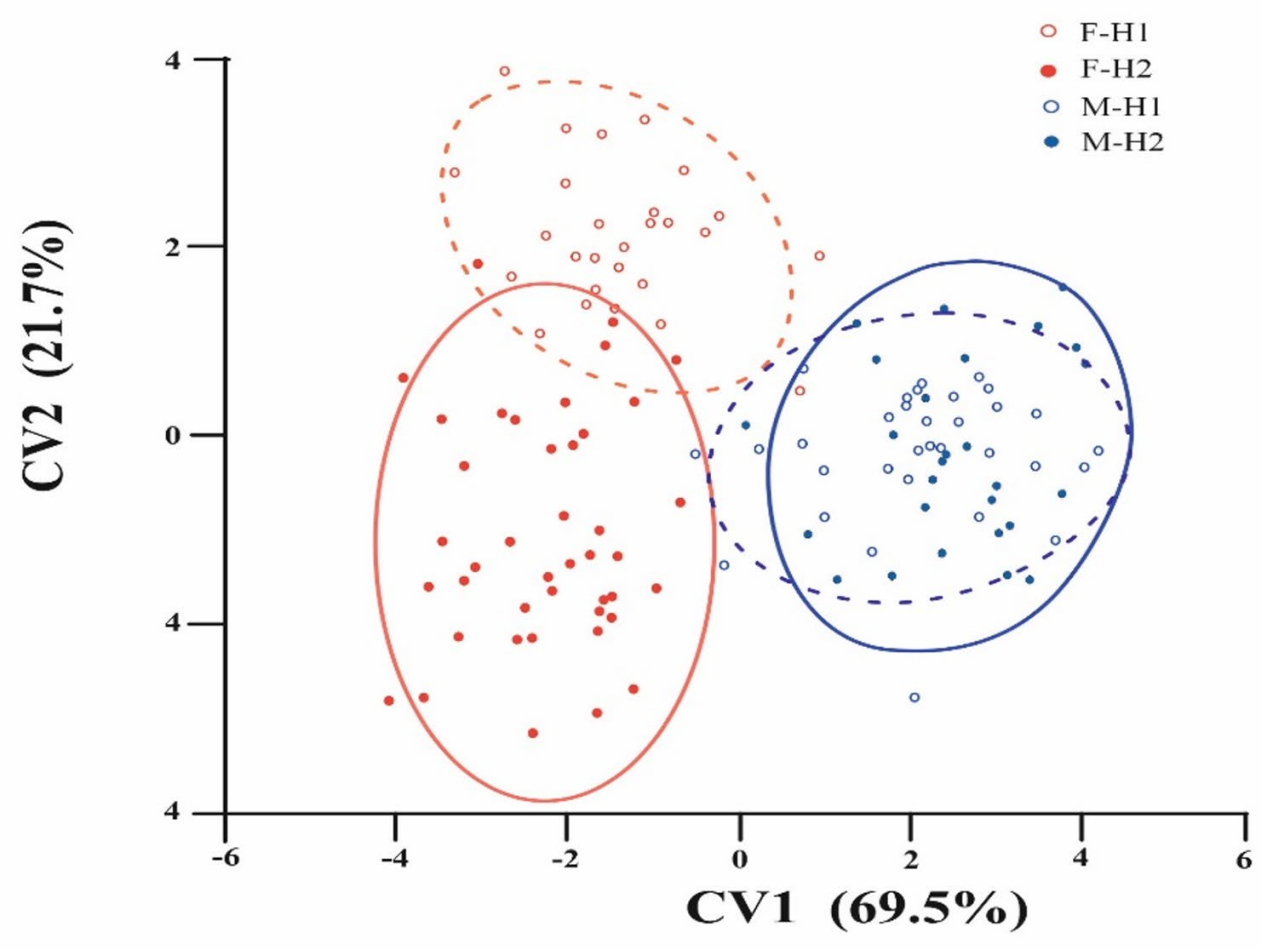
Canonical variate analysis (CVA) illustrating the shape sexual dimorphism between the two populations of spider *Nigma conducens*. The scatter plot illustrates the first two CV axes. H1 = first population, H2 = second population, F and M refer to females and males, respectively

The MANOVA analysis revealed a significant variation in the shape of the prosoma between the pooled sexes (Wilks λ = 1.86, F_(30,98)_ = 14.13, *P* = 0.000), indicating the presence of shape sexual dimorphism in the two populations. Canonical variate analysis (CVA) distinguished the sexes of the two populations into four subgroups, although there was overlap between them, mainly in males (Fig. 7).

CVA yielded three factors that accounted for 100% of the shape variations between females and males. CV1 and CV2 accounted for 69.5% and 21.7% of the total variation, respectively. The arrangement of specimens in morphospace showed that the females of the two populations tended to be on the left side of CVA, while males were on the right side. This arrangement reflects the presence of shape sexual dimorphism between the sexes of both populations, as indicated by significant results of Mahalanobis and Procrustes distances (Table 2). The main deviations in the landmarks are associated with the anterior and posterior regions of the prosoma. Notably, greater shape variation was observed between sexes in the second population.

**Table 2:** Canonical variate analysis (CVA) of shape sexual dimorphism for two populations of *Nigma conducens* spider

Population	Procr. dis.	Mahal. dis.	*T* ^2^	*P* (permutation tests)
H1	0.058	5.23	501.33	< 0.0001
H2	0.087	7.11	684.29	< 0.0001

Procr. dis.= Procrustes distance, Mahal. dis.= Mahalanobis distance, *T*^2^ = Hotelling test

**Fig. 8:**
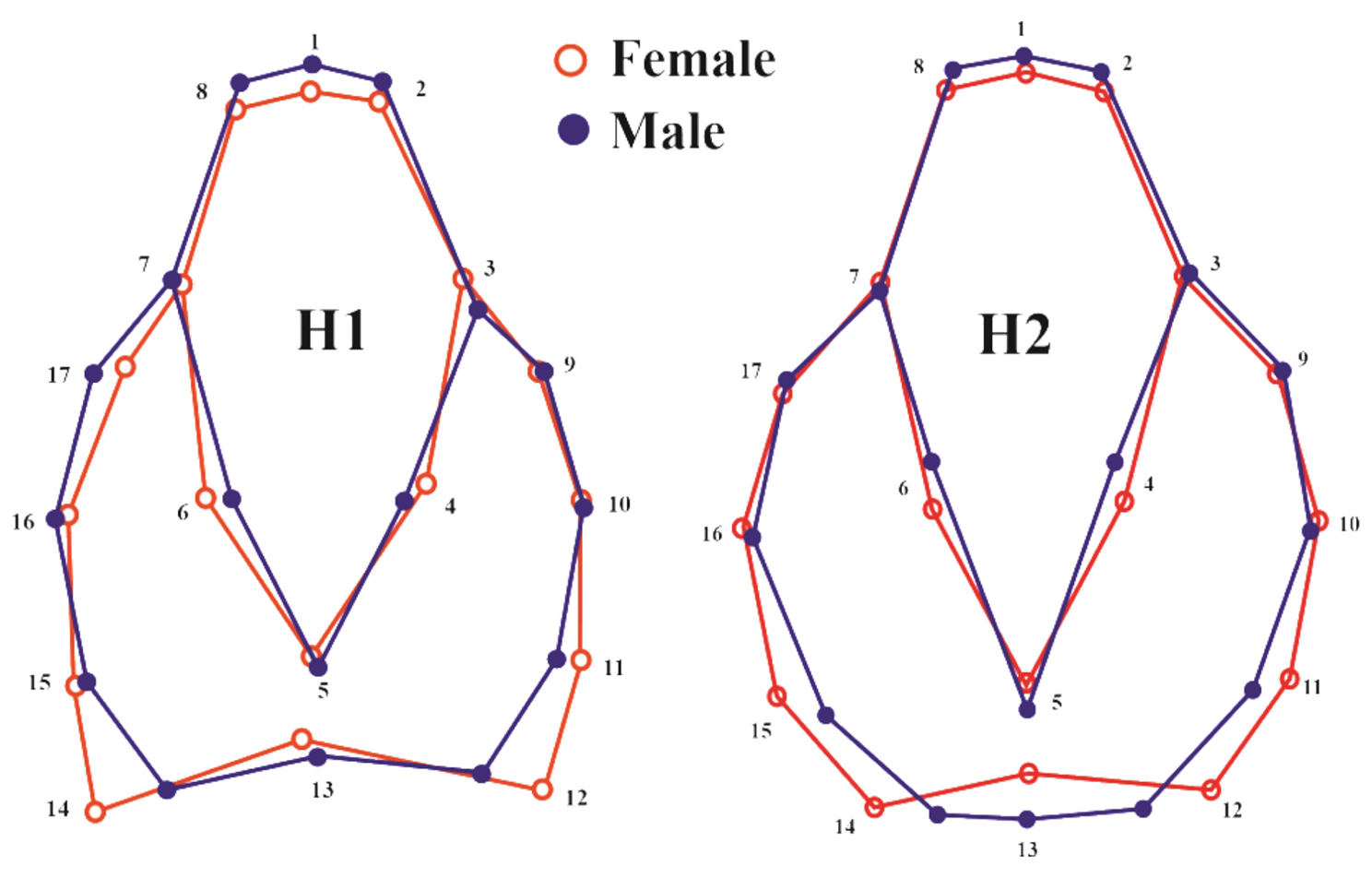
The wireframe diagram illustrates the differences in the mean shapes between male and female prosoma of the spider *Nigma conducens* two populations. The diagram highlights the variation in landmarks between the two sexes. H1 = first population, H2 = second population. (2X magnification to show group differences)

**Fig. 9:**
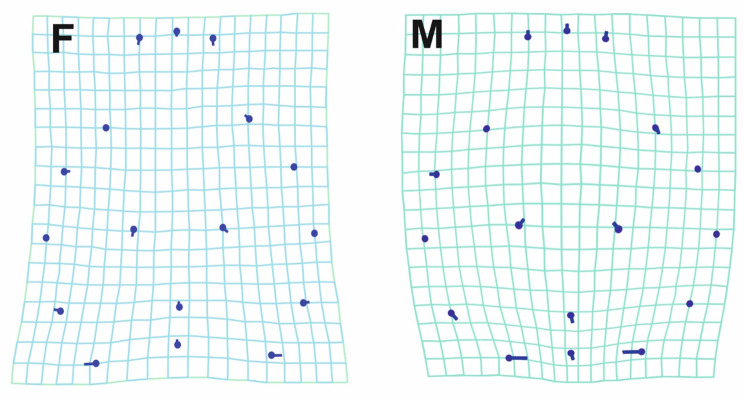
Deformation gride representing the mean shape of female (F) and male (M) of the spider *Nigma conducens* (threefold magnification), showing shape sexual variations in the two parts of prosoma, pars cephalica and pars thoracica. (10X magnification to show group differences)

Females exhibited distinct sexual differentiation in both populations compared to males, with a slightly shortened anterior region and wider pars thoracica (Fig. 8). In the posterior region of pars thoracica, the differences were more pronounced in the second population: females displayed a lateral expansion extending from the middle to the posterior end of the prosoma. In contrast, this expansion in females of the first population was restricted solely to the posterior region. Furthermore, the posterior region of the pars thoracica in the second population’s females was shorter than that of males. The resulting deformation gride indicates that males are characterized by elevated pars cephalica, which was nearly flattened in females (Fig. 9).

DFA supported the results of CVA (Fig. S2) for the sexes. DFA correctly classified 100% of the individuals based on sex, while cross-validation analysis achieved 89% accuracy in classification.

Allometric analysis for the sexes of each population revealed no effect of size on shape as shown by interaction (Table 3).

**Table 3:** Multivariate regression analysis (MANCOVA) of prosoma shape of *Nigma conducens* spider on log centroid size

Population	Effect	λ	F	df1	df2	*P*
H1	sex	0.63	0.88	30	42	0.72
	Sex X logCs	0.38	0.84	30	42	0.68
H2	sex	0.41	0.87	31	19	0.86
	Sex X logCs	0.58	0.86	30	42	0.65

H1 and H2 referred to the two populations

Allometric analysis for females in each population revealed no effect of size on shape (permutation test *P* = 0.07). On the other hand, males in both populations showed a little allometric effect (permutation test *P*_H1_=0.038; *P*_H2_=0.041), accounting for 2.3% and 4.3% of the total variation, respectively.

## Discussion

The present study compared two populations of *Nigma conducens* spider in terms of their prosoma size and shape, as well as the size and shape of sexual dimorphism using the landmark geometric morphometrics technique. The results illustrate that (1) two populations differ slightly in their prosoma shape and size, (2) shape variations are non-allometric in two populations with minimal value, (3) the prosoma shape of sexes is sexually dimorphic and slightly independent of size, while prosoma size is not sexually dimorphic.

The GMM revealed prosoma shape variations between the two populations as illustrated by MANOVA and DFA. The first population had a slightly protruded cephalic region and narrowed lateral sides, while the second population had expanded lateral sides and a slightly reduced cephalic region. Data exploration through PCA revealed that variations in prosoma shape between the two populations were insufficient to distinguish them, indicating that they are not morphologically distinct. This overlap in prosoma shape reflects a lack of divergence between the populations and contributes to the large percentage of misclassification of individuals to their respective groups. Such a large degree of morphological similarity may stem from the shared characteristics of their microhabitats, which exert somewhat similar ecological pressures on the populations.

Environmental factors, particularly weak ecological pressures, may play a key role in driving this morphological convergence. The limited geographic scope of study sampling may have also contributed to the observed lack of variation, as it may not fully capture differences across the species’ broader geographic range [51]. The lack of distinct habitat variability significantly reduces the morphological diversity of spiders. In homogeneous environments, stable and predictable conditions limit the availability of diverse niches, leading to convergence toward similar/partially similar shapes that perform well across uniform challenges. These morphologies allow spiders to efficiently exploit consistent resources while reducing the need for pronounced morphological variations.

In heterogeneous environments, ecological niches drive the evolution of distinct morphologies, but in uniform settings, these pressures are less pronounced. Behavioral adaptations can sometimes compensate for the lack of specialized traits, allowing species to thrive without significant morphological changes [52]. Ultimately, homogeneous conditions favor versatility over specialization, stabilizing traits that promote general functionality and resilience while narrowing the scope for morphological diversity.

The large degree of phenotypic similarity observed between the prosoma of the two populations can be attributed to their comparable microhabitats. Both populations occupy environments where the leaves of the host trees share many features such as leathery textures, glossiness, and oval shapes, providing suitable habitats for insects that serve as prey for the spiders [53]. This suggests that *N. conduce* populations inhabit almost similar ecological niches, and their geographical distribution does not strongly correlate with distinct morphological patterns [54, 55]. In this regard, considering the microhabitats of spiders are partially homogeneous environments, where selective pressures are relatively uniform, spiders may evolve streamlined and less varied shapes suited to the consistent environmental demands [51, 56].

Morphological traits, such as the prosoma shape, reflect a balance between environmental constraints and evolutionary pathways [57]. The large degree of similarity in the prosoma phenotype may represent an adaptive response to shared selective pressures in these habitats through natural selection. Alternatively, it may act as an exaptation that enhances fitness in these environments [52]. This convergence suggests that the phenotype results from the interaction between genotype and habitat conditions. Given the apparent lack of significant geographic or environmental isolation between the two populations, it is plausible that their genotypes remain similar and have not diverged significantly through isolation [58].

The river Nile works as a geographical barrier between the two populations and can limit gene flow and promote divergence. However, the occurrence of morphological changes is often shaped by the complex interplay of ecological, genetic, and evolutionary factors. In this study, the maintenance of shape similarity despite physical barriers reflects nearly similar microhabitats, weak selective pressures, and a common genetic or developmental architecture [59]. Also, the two populations of *N. conduce* are historically connected and may recently be separated, which means that they still retain shared morphological traits due to their common ancestry [60].

The observed variations in prosoma shape can be linked to the functional roles of prosoma in adult spiders, which include locomotion, food acquisition, and nervous integration [61]. These variations may have an evolutionary basis, potentially reflecting adaptations to differing locomotory and feeding strategies. Such adaptations could enhance prey-capturing efficiency and may also be influenced by distinct growth patterns, further shaping prosoma morphology [62]. This suggests that prosoma shape variations are not arbitrary but result from selective pressures that optimize survival and resource acquisition in different environments.

Environmental factors, including agrochemical exposure, may also play a significant role in shaping spider morphology. For instance [32], reported that agrochemical treatments affected the morphology of female *Oedothorax apicatus* spiders but not males. Similarly, in the present study, the trees in the second site, surrounding an agricultural field exposed to pesticide applications, may have experienced both direct and indirect impacts on spider morphology. Research shows that low doses of certain pesticides can sometimes have unintentional beneficial effects, such as increasing growth rates and body size in non-target organisms [63, 64]. These findings highlight the complex interactions between environmental stressors and morphological traits.

Additionally, shape variations between individuals from the two populations may reflect enhanced foraging abilities, which could ultimately lead to greater body growth [65]. This is particularly relevant in the context of insecticide use, as studies have shown that prey activity often increases after insecticide application [66]. For spiders in the first population, this could result in greater prey availability, leading to a higher growth rate and potentially influencing their prosoma morphology. Thus, the interaction between environmental conditions, prey dynamics, and evolutionary pressures likely drives the observed phenotypic differences between populations.

In terms of morphology, allometry plays a significant role in the overall structure and organization of an organism’s phenotype. Understanding allometric variation is essential for examining morphological differences and similarities, including those in the prosoma. However, in the present study, shape changes in the prosoma were found to be independent of size, indicating that the observed variations were not allometric in nature. This suggests that differences in size between the two populations did not influence their prosoma shapes. The slight size difference observed between the populations may further explain the lack of a strong allometric relationship and the overlap in shape variation noted in this research.

Ontogenetic allometries often contribute to the variation observed in adult allometric traits. These traits can then undergo modifications over evolutionary time, shaping the morphological diversity of species [67]. However, the findings of this study suggest that ontogenetic allometry did not contribute significantly to prosoma shape differences in the populations examined. Instead, the variations in shape may be shaped by factors other than size.

### Sexual dimorphism

The present study examines sexual dimorphism in the prosoma of *N. conducens* spiders, focusing on size and shape while excluding the abdomen. The abdomen, highly variable in size and shape due to its role in fecundity, is subject to evolutionary pressures that increase volume, weight, and shape for reproductive purposes [33, 68–70]. By concentrating on the prosoma, a more stable morphological trait, the study offers a clearer understanding of how sexual selection and microhabitat features influence dimorphism.

The results reveal that *N. conducens* spiders exhibit sexual dimorphism in the shape of the prosoma but not its size. This finding is supported by CVA analysis and significant Mahalanobis and Procrustes distances. Sexual dimorphism in spiders varies widely among species, with some showing marked differences between males and females and others remaining similar in size and shape [71]. Importantly, shape differences in spiders generally emerge after the final molt, as most species exhibit determinate growth and do not molt beyond maturity [61]. Thus, the adult prosoma reflects a fixed morphological trait, which, if dimorphic, provides a window into underlying evolutionary processes.

The absence of size dimorphism in *N. conducens* may be explained by the comparable sizes of the specimens studied or by the fact that the prosoma lacks a direct role in fecundity. This uniformity in prosoma size between sexes likely reflects a plesiomorphic (ancestral) trait preserved within the species [68].

Size dimorphism in spiders is typically studied using traditional metrics, such as total body length, which includes both the prosoma and abdomen [72–74]. However, this trait is highly variable across species, with some exhibiting larger females, others larger males, and many showing no significant size differences at all [33, 75].

In terms of shape dimorphism, females of *N. conducens* show subtle differences in prosoma morphology. In the first population, females show a lateral expansion only in the posterior part of pars thoracica, whereas in the second population, the expansion extends from the middle to the posterior region. Also, females in the second population display a shorter posterior pars thoracica compared to males. Meanwhile, the posterior expansion in females aligns with early adaptations for reproduction, such as the gradual widening of the abdomen. Also, subtle differences in males (slight frontal protrusion in the prosoma and elevated pars cephalica) may be linked to the size of paired pedipalps used for grasping or holding females during copulation [61]. These variations in prosoma shape may reflect ecological pressures specific to different microhabitats, suggesting that the forces driving dimorphism vary between populations.

If sexual selection were the sole factor driving the observed dimorphism, no additional differences in prosoma shape would be expected [76]. However, Herrel et al. [77] hypothesized that sexual shape dimorphism may also result from niche differentiation between sexes. In the current study, males and females were found on different leaves of the same tree, often adjacent but sometimes further apart. This spatial separation supports the idea of niche differentiation as an adaptive strategy, reducing competition for resources while allowing both sexes to optimize their reproductive and survival roles.

The subtle shape dimorphism observed in *N. conducens* may indicate that niche differentiation is more focused on ecological factors, such as microhabitat use or prey selection. This reduced selective pressure for distinct morphological adaptations, such as body shape differences, aligns with the observed minor shape differences. Males may prioritize rapid growth for mobility, while females invest more energy in reproduction, potentially explaining the widening of the female prosoma’s posterior region.

The weak shape sexual dimorphism in the prosoma of *N. conducens* suggests a limited role for sexual selection in shaping this trait [78]. In contrast, other spiders, such as the European orb-weaving spider *Metellina segmentata*, exhibit more pronounced shape dimorphism, with males having broader prosoma [23]. At the genus level, the evolution of size and shape dimorphism in orb-weaving spiders often correlates strongly, with some lineages showing extreme dimorphism where females are significantly larger than males.

Overall, the findings indicate that the prosoma contributes modestly to sexual shape dimorphism in *N. conducens*. The observed differences likely result from a combination of reproductive strategies, ecological pressures, and niche differentiation rather than solely from sexual selection. This complex interplay underscores the importance of considering both morphological and ecological perspectives when studying sexual dimorphism in spiders.

## Conclusion

The present study uses GMM analysis to examine prosoma size and shape in two spider populations of *Nigma conducens*, revealing insights into the evolutionary and ecological factors influencing their morphology. Slight differences in prosoma shape and size were found between populations, with shape variations being non-allometric and sexually dimorphic, while size dimorphism was absent. Shared microhabitats and weak selective pressures likely maintain the morphological similarity between populations, despite geographical barriers like the River Nile. Shape variations appear to reflect subtle adaptations to ecological and functional demands rather than significant evolutionary divergence. The environmental uniformity of habitats, characterized by partially similar host tree features and prey availability, likely drives this morphological convergence. Historical connections and recent separation may also have limited genetic divergence, preserving shared traits. Weak sexual dimorphism in prosoma shape suggests niche differentiation and sex-specific roles, with males showing slight frontal protrusions and females displaying posterior expansions potentially linked to reproduction. Ecological factors, including microhabitat use and prey dynamics, may primarily drive this dimorphism. This study highlights the role of environmental characteristics in shaping spider morphology and enhances our understanding of the adaptive strategies in *N. conducens*.

## Electronic supplementary material

Below is the link to the electronic supplementary material.

Supplementary Material 1

Supplementary Material 2

Supplementary Material 3

## Data Availability

No datasets were generated or analysed during the current study.
