# Supplementary material for: Exploring shape diversity and sexual dimorphism in two populations of Nigma conducens through geometric morphometrics
Source: BMC Zool. 2025 Feb 8;10:4. doi: 10.1186/s40850-025-00224-4 (PMC11806776; doi:10.1186/s40850-025-00224-4)
Supplement: Supplementary file 1 — Supplementary Material 1 [file 40850_2025_224_MOESM1_ESM.doc]

**Table S2**

Measurement errors of landmarks digitization of *Nigma conducens* spider in two populations using Procrustes ANOVA. H1 and H2 referred to the two populations.

Effect % Explained

Shape variance SS MS df *F* *P* (param.) Pillai tr. *P* (param.)

H1 Individuals 99.94 0.44620 0.000320 1394 199.6 <0.0001 33.71 <0.0001

Digit. error 0.060 0.00023 0.000002 1428

H2 Individuals 99.85 0.29235 0.000330 884 678.6 <0.0001 29.25 <0.0001

Digit. error 0.51 0.00044 0.000002 918
