# Supplementary material for: Exploring shape diversity and sexual dimorphism in two populations of Nigma conducens through geometric morphometrics
Source: BMC Zool. 2025 Feb 8;10:4. doi: 10.1186/s40850-025-00224-4 (PMC11806776; doi:10.1186/s40850-025-00224-4)
Supplement: Supplementary file 2 — Supplementary Material 2 [file 40850_2025_224_MOESM2_ESM.doc]

**Table S1**

The main features of the *Dalbergia sissoo* and *Ficus nitida* trees are inhabited by the *Nigma conducens* spider.

Features *Dalbergia sissoo* *Ficus nitida*

Canopy Density Open Dense

Leaf Arrangement Alternate Alternate

Leaf Venation Brachidodrome Pinnate

Leaf Type Pinnate Simple

Leaf Shape Oval Oval

Leaf Surface texture Smooth and slightly leathery Smooth and leathery

Leaf Thickness Moderately thick Thick

Leaf Glossiness Moderately glossy Highly glossy

Leaf Color Medium green Dark green

Leaf Persistence Deciduous Evergreen
