# Supplementary material for: Exploring shape diversity and sexual dimorphism in two populations of Nigma conducens through geometric morphometrics
Source: BMC Zool. 2025 Feb 8;10:4. doi: 10.1186/s40850-025-00224-4 (PMC11806776; doi:10.1186/s40850-025-00224-4)

**Fig. S1.** Images showing the morphology of trees host spider; A) *Ficus nitida* in the first site; B). *Dalbergia sissoo* in second site.


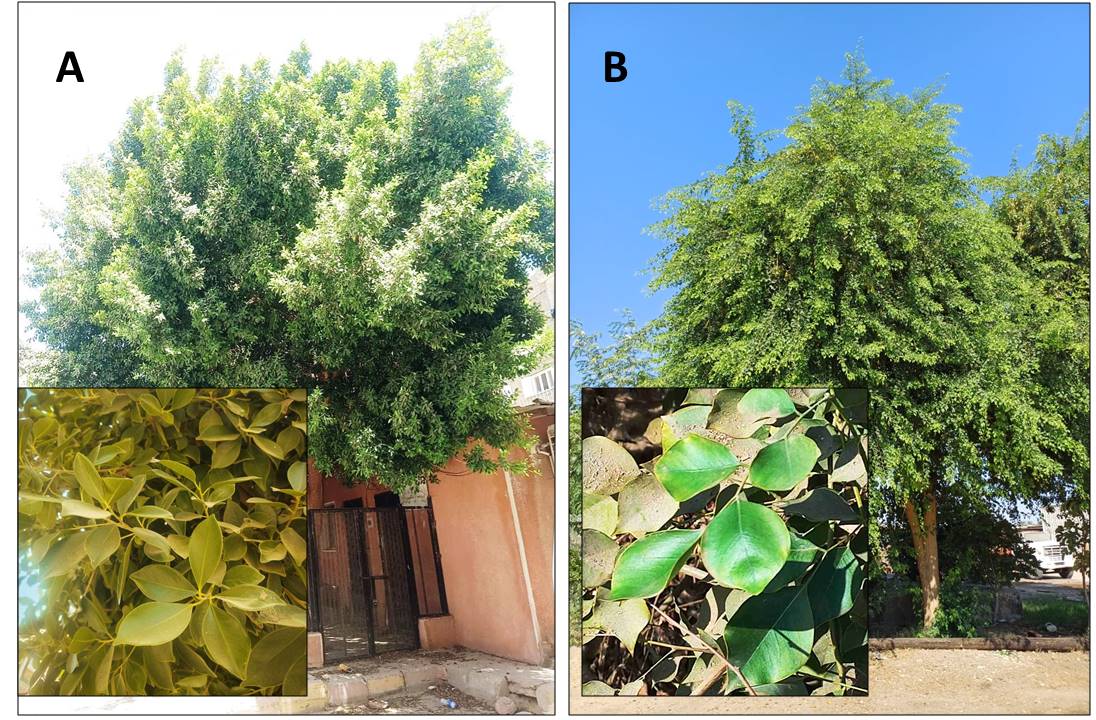


**Fig. S2.** Discrimination functional analysis (DFA) showing that female and male shapes can be distinguished within each population.


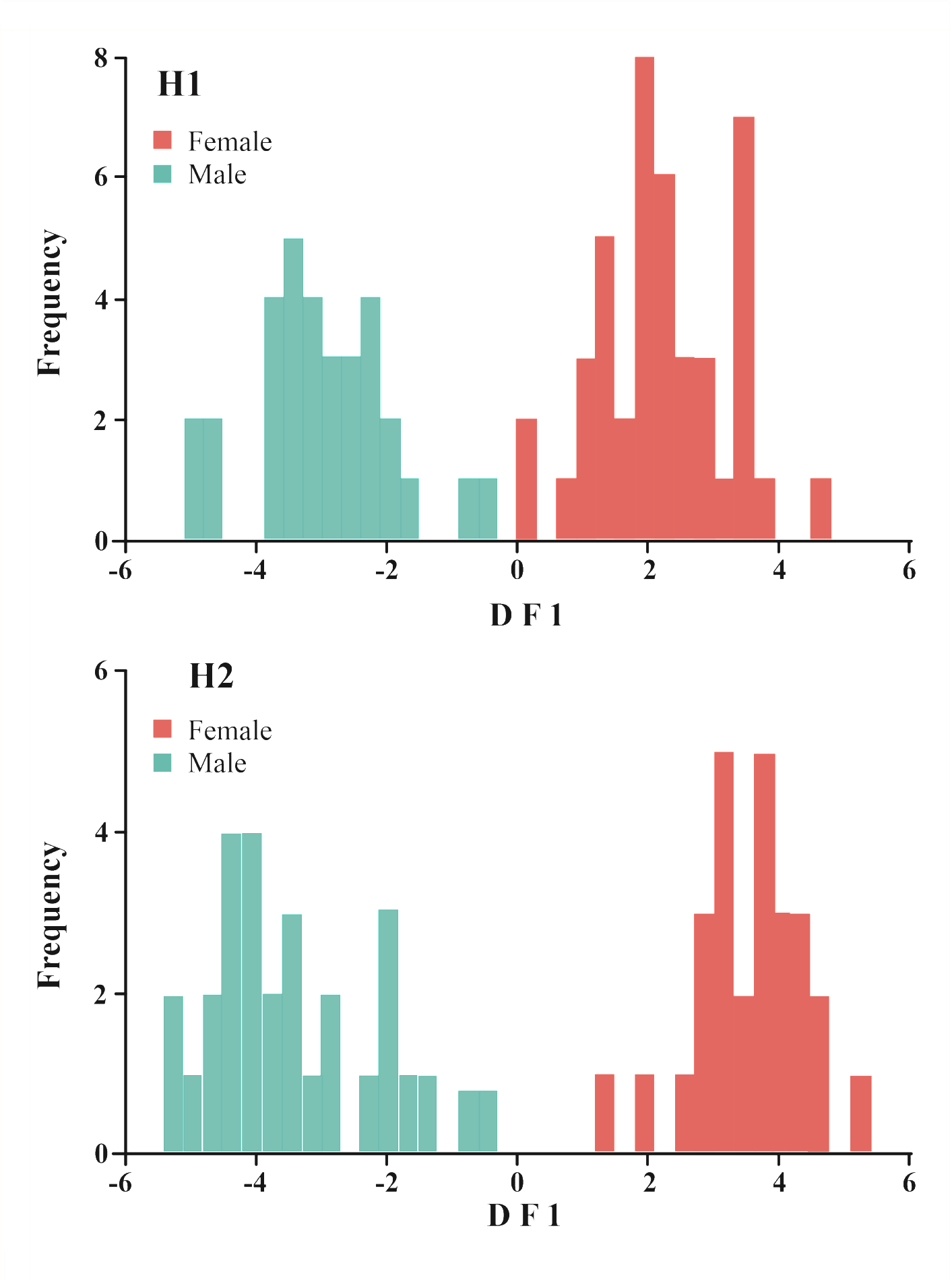

Supplement: Supplementary file 3 — Supplementary Material 3 [file 40850_2025_224_MOESM3_ESM.docx]
